# Supplementary material for: Learning curve and surgical outcome of robotic assisted colorectal surgery with ERAS program
Source: Sci Rep. 2022 Nov 29;12:20566. doi: 10.1038/s41598-022-24665-w (PMC9709162; doi:10.1038/s41598-022-24665-w)
Supplement: Supplementary file 1 — Supplementary Information 1. [file 41598_2022_24665_MOESM1_ESM.zip › LSC Raw data-Ñ[▒K/077/H_ñw╝╨░O▒Kñσ.pdf]

正式摘要(電子簽章：000000189848734)

W83 / W83 ☐ 英文標題(注意:無英文姓名-請補病人基本檔!) ☐【讀取】憑證

臺中榮民總醫院
出院病歷摘要

轉入號 : 轉出號 :

[redacted]

(8)流水編號	
(9)入院日期	109/04/21 (2020) 直腸外科 W83 - 052
(10)轉科日期	
(11)出院日期	109/04/28 (2020) 住院天數計 7 日

診斷

- 入院 :1.S-Colon Cancer, c T3 N0 M0, Stage IIA
- 出院 :1.S colon adenocarcinoma, s/p laparoscopic LAR on 2020/04/22 pT3N0(0/32)M0, Stage IIA, MSS. MD

主訴

- incidential finding of S colon tumor

病史

- This 60-year-old female with history of
  1. Gallbladder stone s/p laparoscopic cholecystectomy 2019/05/07.
  2. Acute appendicitis s/p appendectomy in 2008.
  According to the patient's statement, her husband was diagnosed colon cancer 2 years ago. Her FOBT result was normal before. There's no hematochezia, no abdominal pain, no change of bowel habit, no body weight loss. Under the doctor's suggestion, she received colonscopy which S colon adenocarcinoma 20 cm FAV was found. Further tumor survey showed no distant metastasis. Under the impression of Colon Cancer, c T3 N0 M0, Stage IIA , he was admitted for scheduled laparoscopic LAR.

體檢發現

- COS: to cecum, s/p Px A colon*2.
  S colon lesion 20 cm FAV s/p biopsy.
  - General appearance :
    - easy looking , conscious clear , without cardiopulmonary distress
    - Vital signs自動帶入date 體溫(BT) 脈搏(P) 呼吸(R) 血壓(BP) 身高(BH) 體重(BW) BMI
      20200421 35.4℃ 74次/分 18次/分 125/66mmHg 151cm 54kg 23.7
    - Vital signs: B.P. 125/66 mmHg , P.R. 74 /min , R.R. 18 /min , B.T. 35.4 ℃
  - HEENT :
    - Eyes: no anemic conjunctiva, no icteric sclera, normal EOM
    - ENT: no otorrhea, no rhinorrhea, no erythematous mucosa, no injected tonsil, no deformity
  - Neck: supple, freely movable, trachea midline,no deformity, no engorged jugular vein , no palpable thyroid or lymph node enlargement
  - Chest:
    - Inspection: Grossly normal, symmetric and fully expansion, no accessory muscle used, no operation scar, no spider angiomata, no gynecomastia
    - Palpation: no palpable mass or nodule, no subcutaneous emphysema, no tenderness
    - Ausculation: clear breathing sound
  - Heart:
    - Palpation: no thrill, no heave
    - Percussion: normal heart size
    - Ausculation: regular heart beat, normal S1, S2, no gallop, no murmur
  - Extremities: No deformity, full ranged of motion without limitation, no clubbing finger or nail cyanosis, no pitting edema, no ulcer or pigmentation, no obvious skin lesion,

- no varicose vein, no bone, joint or muscle tenderness

CRS CONDITION

- Karnofsky performance status: (100) Normal, no complaints, no evidence of disease
- PE of Abdomen:
- CEA: pending ng/ml
- Imaging Studies:
- Colonoscope report: 活體組織切片病理報告 手術日期：109/04/07 病理號：S0909886 診斷醫師:吳雅茹 報告日期:109/04/13 Pathologic diagnosis: Colon, "tumor", 20 cm from anal verge, endoscopic biopsy --- Adenocarcinoma, moderately differentiated. Ancillary study for diagnosis: 1. Step cut x 3 done. 2. AE1/AE3 (+) immunohistochemical stain done. Gross description: The specimen consists of 4 pieces of tan soft tissue, up to 0.5x0.3x0.3 cm. All for section. Microscopic description: Section shows colon mucosa with moderately differentiated invasive carcinoma with glandular differentiation. Comment: Further clinicopathological correlation is recommended. Note: The diagnosis is concurred in intradepartment consensus meeting on 2020/04/13. #T-59470_2 #M-81403_2 2 1340 00CM0F
- Figures:
  1. colon & rectum

手術日期及方法

- 手術日期及方法20200422 Laparoscopic Anterior resection and anastomosis - malignant

住院治療經過

- After admission, laparoscopic LAR was performed on 2020/04/22. After removing foley and NG, she tried food smoothly. Penrose and stiches were also removed. Under stable condition, she was discharged with OPD f/u

合併症

一般檢查

- 累積報告 :

DATE	TIME	ALB	TP	BIL.T	BIL.D	ALKP	AST	ALT	LDH	CHOL	TG	HDL-C	LDL	UA	NH3	AMY
1090421	1829	5.0		0.7	0.1	79	49	50	230							
1090423	0906	4.1		0.6				39								
1090427	1215	4.4		0.5			26	17								

DATE	TIME	WBC	RBC	HGB	HCT	MCV	PLT	BLST	NEUT	BAND	SEG	LYM	MON	EOS	BAS
1090421	1828	7680	4.73	14.2	42.9	90.7	223		55.1			37.2	6.0	1.0	0.7
1090423	0907	13200	4.46	13.5			196		76.3			16.9	6.6	0.0	0.2
1090427	1213	9040		14.0			153		69.3			20.9	6.3	3.1	0.4

DATE	TIME	NA	K	CL	CA	BUN	CREAT	Mg	P	CRP	ESR	PCT	LIPASE	LACTATE
1090421	1829	143	4.1	104	9.2	12	0.60							
1090423	0906	141	4.6	105	8.5	5	0.64							
1090427	1215	140	4.1		8.8	7	0.46							

DATE	TIME	GLUCOSE	PAB	TRF	ZN	CU	KETONE	CK	CKMB	PT-P	PT-C	APTT-P	APTT-C	TRO-T
1090421	1827									10.6	10.3	28.0		
1090421	1828	92												

特殊檢查

- **檢驗項目 :CT, LUNG 報告日期 :1090416**

主　訴：

Result:

Finding:

CT of chest with IV contrast enhancement.

1. Sigmoid colon cancer for study.

2. No significantly abnormal nodular lesion in bilateral lungs, or lymphadenopathy in the mediastinum.

3. No cardiomegaly.

Mild atherosclerosis of thoracic aorta.

4. Mild fatty liver.

No abnormal focal lesions in the liver.

No hepatosplenomegaly.

Impression:

As above description.

- **檢驗項目 :CT ABDOMEN-SURVEY 報告日期 :1090414**

主　訴：
Result:
Finding:
1. Imaging Modality and protocol:
CT of abdomen with IV and oral contrast.
2. Tumor location and size:
R/O sigmoid colon Ca; The tumor location and size are difficult to be determined.
- mottling feces-gas pattern in the course of sigmorectal colon,
3. Tumor invasion:
Slightly dirty pericolic fat planes.
4. Regional lymph node metastasis:
Not assessable
5. Distant metastasis:
No evidence of abnormal lymphadenopathy in paraaortic
space.
6. Other findings:
> fatty infiltration of liver
> S/p cholecystectomy
> the appearance of liver, spleen, pancreas and left kidney is not unusual,
> right renal cyst
> wall thickening of ascending and transverse colon with enhancing mucosa, in favor of colitis.
Impression:
> Sigmoid colon Ca, for staging.

檢驗項目 :CHEST PA VIEW 報告日期 :1090415

主　訴：
Result:
Finding:
Mild tortuosity of T-aorta.
No cardiomegaly.
Honey-comb appearance and chronic interstitial infiltration over both lungs.
Impression:
As above description.

檢驗項目 :ANTI-HCV 報告日期 :0980629

RESULT　　CUTOFF VALUE
* 0.087　*　* 0.680　*
* Negative　*　*　　　　*
(POSITIVE:>1.0)
(NEGATIVE:1.0)
COMMENT:

檢驗項目 :HBsAg 報告日期 :0980626

RESULT　　CUTOFF VALUE
* 0.359　*　*　1　　*
* Negative　*　*　　　　*
(POSITIVE:>1.0)
(NEGATIVE:1.0)
COMMENT:

病理報告

- 活體組織切片病理報告　手術日期：**109/04/07**　病理號：**S0909886**

診斷醫師:吳雅茹 報告日期:109/04/13
Pathologic diagnosis:
Colon, "tumor", 20 cm from anal verge, endoscopic biopsy ---
Adenocarcinoma,
moderately differentiated.
Ancillary study for diagnosis:
1. Step cut x 3 done.
2. AE1/AE3 (+) immunohistochemical stain done.
Gross description:
The specimen consists of 4 pieces of tan soft tissue, up to
0.5x0.3x0.3 cm. All for section.

- Microscopic description:
  Section shows colon mucosa with moderately differentiated invasive carcinoma with glandular differentiation.
  Comment:
  Further clinicopathological correlation is recommended.
  Note:
  The diagnosis is concurred in intradepartment consensus meeting on 2020/04/13.
  #T-59470_2 #M-81403_2 2 1340 00CM0F

活體組織切片病理報告　手術日期：**109/04/22**　病理號：**S0911205**

診斷醫師:陳韻安 報告日期:109/04/28
Pathologic diagnosis:-
1. Sigmoid colon, laparoscopic anterior resection --- Adenocarcinoma, moderately differentiated. Tumor invaded to muscularis propria. The bilateral cut ends and mesocolic lymph nodes (0/28) are free of tumor.
2. Colon, cut end, distal, resection --- Hyperplastic polyps.
3. IMA root, dissection --- Unremarkable (0/4).

Ancillary study for diagnosis:
1. Deep cut done in section G and H.
2. IHC stains for MLH1, MSH2, PMS2 and MSH6 are done on section H. All above markers are preserved.

Prognostic and predictive factor:
1. Tumor size: 2x2.4 cm.
2. Depth of invasion: Muscularis propria.
3. Histologic grade: G2: Moderately differentiated.
4. Angiolymphatic invasion: Not identified.
5. Perineural invasion: Not identified.
6. Tumor deposits (discontinuous extramural extension): Not identified.
7. Macroscopic tumor perforation: Not identified.
8. Surgical Margins:
a) Proximal Margin: Uninvolved by invasive carcinoma, distance of tumor from margin: 6.5 cm.
b) Distal Margin: Uninvolved by invasive carcinoma, distance of tumor from margin: 3.8 cm.
c) Radial or Mesenteric Margin: Uninvolved by invasive carcinoma, distance of tumor from margin: 5.8 cm.
9. Lymph node status: Uninvolved (0/32).
10. Extranodal involvement: Not applicable.
11. Tumor regression grading S/P CCRT: No known presurgical therapy.
12. Type of polyp in which invasive carcinoma arose: Not applicable.
13. Additional pathologic findings: Hyperplastic polyp.
14. Pathological TNM Stage: pT2N0(According to the eighth edition, American Joint Committee on Cancer Staging Guidelines for Tumors).
15. TNM descriptors: Not applicable.

Gross description:
The specimen consists of 1) a segment of colon, 13 cm in length and 5.8 cm in proximal cut end circumference, 5 cm in distal circumference, and mesocolic soft tissue. A gray tan firm annular elevated and centrally ulcerative tumor, 2x2.4 cm, is noted, 3.8 cm to the nearest cut end. On cross section, the tumor invades to the muscular layer with 5.8 cm to the radial margin. The both cut ends are free of tumor grossly. Besides, some small polyps, up to 0.4x0.2x0.2 cm, are noted among the colon. 2) distal cut end, a piece of tan mucosa-covered soft tissue, 1.5x1x0.8 cm. 3) IMA root, a piece of tan yellow soft tissue, 5.5x3x1 cm. All for section: A) specimen 2 B-C) specimen 3; Representative part for section: D) proximal cut end E) radial cut end F-H) tumor J) polyps K-M) lymph nodes.
Note:
1. The interpretation of MMR panel and recommended studies are as below:
(1) No loss of nuclear expression of MMR proteins: low probability of microsatellite instability-high (MSI-H).
(2) Loss of nuclear expression of MLH1 and PMS2: testing for methylation of the MLH1 promoter and/or mutation of BRAF is indicated

- (the presence of a BRAF V600E mutation and/or MLH1 methylation suggests that the tumor is sporadic and germline evaluation is probably not indicated; absence of both MLH1 methylation and of BRAF V600E mutation suggests the possibility of Lynch syndrome, and sequencing and/or large deletion/duplication testing of germline MLH1 may be indicated).
(3) Loss of nuclear expression of MSH2 and MSH6: high probability of Lynch syndrome (sequencing and/or large deletion/duplication testing of germline MSH2 may be indicated, and, if negative, sequencing and/or large deletion/duplication testing of germline MSH6 may be indicated).
(4) Loss of nuclear expression of MSH6 only: high probability of Lynch syndrome (sequencing and/or large deletion/duplication testing of germline MSH6 may be indicated).
(5) Loss of nuclear expression of PMS2 only: high probability of Lynch syndrome (sequencing and/or large deletion/duplication testing of germline PMS2 may be indicated).
(6) Please note: There are exceptions to the above IHC interpretations. These results should not be considered in isolation, and clinical correlation with genetic counseling is recommended to assess the need for germline testing.
2. This case has been peer reviewed by two doctors.
#T-59472_2 #M-81403_2 2 1105 M0000F

其它

出院時情況

- 3.改門診治療

出院指示

藥名	劑量	劑量單位	用法	頻次	數量	數量單位	現況	天數	單位
(口)Takepron OD tab 30mg	1	TAB	PO	QDAC	7	TAB	TH	7	天
Ultracet tab	1	TAB	PO	Q6HPRN	28	TAB	TH	7	天

出院計劃

主治醫師 2222H 林俊余 / 住院醫師 5738B 方姿貽

正式摘要(電子簽章：000000189848734)

W83 / W83

doctorRank:B
